# Obituary

**Published:** 1876-06

**Authors:** 


### OBITUARY.

Dr. William W. Hall, the founder and editor of the Journal of Health, died suddenly on the evening of May 10th. During thirty years he had not been ill a day. He had nearly attained his three score and ten years. In spite of a naturally frail physical organization, he might have attained an additional score of years. But few of the readers of the Journal of Health had ever seen the man whose name for a quarter of a century was a household word in thousands of intelligent families. Any impressions in regard to the personal appearance of Dr. Hall, based upon his strong and vigorous style as a writer, are likely to be erroneous. In stature he did not exceed five feet and a half, and he was quite slender. In manners he was modest and unobtrusive, of pleasing address, and musical, earnest voice, which added force to his reasoning.

His death was instantaneous, as he had always foretold and desired. That it was occasioned by overwork, can not be doubted. His one and only dissipation was labor. It was no unusual thing for him to labor in his office for eighteen hours of the twenty-four. He took little exercise, because he had, or thought he had, no time to indulge in it. He achieved results because he labored untiringly. His published volumes number about thirty, exclusive of the twenty volumes of the Journal, which he edited without assistance. He was an able counsellor in matters pertaining to health, but failed to live according to the precepts which he uttered. Like other guide-boards, he pointed the way through pleasant paths in which he never walked.

The writings of Dr. Hall have attained great popularity. His books have sold to the extent of hundreds of thousands, but he regarded the Journal of Health as his great life work. It was the first and most successful effort ever made in any country to establish a Journal devoted to instructing the people so to live as to attain the largest measure of health and happiness.

Dr. Hall's last book bore the title "How to Live Long," and was a series of maxims, the observance of which on his part would probably have tended to prolong his life for many years. The constant violation of the very laws which he so nobly expounded, unquestionably accounts for his sudden taking off.

Dr. Hall had a very extensive acquaintance throughout the country, and his unexpected demise will be widely and sincerely mourned. Upon none will the loss fall more heavily than upon his co-adjutors in the management of the Journal, upon whom the active labor of its publication long since devolved, and who will continue it in accordance with its founder's frequently expressed wish.

